# The impacts of quantity and quality of health clinics on health behaviors and outcomes in Nigeria: analysis of health clinic census data

**DOI:** 10.1186/s12913-019-4141-y

**Published:** 2019-06-13

**Authors:** Ryoko Sato

**Affiliations:** Harvard T.H. Chan School of Public Health, 90 Smith St, Boston, MA 02120 USA

**Keywords:** Health services, Quality of care, Health care utilization, Nigeria

## Abstract

**Background:**

Past studies have identified that inconvenient access to health clinics is one of the important barriers to health service utilization and health outcomes. However, establishing the link between the lack of access to health clinics and the high maternal and child morbidity and mortality in Nigeria has been a challenge due to the lack of data. This paper overcomes this problem by using the country’s health clinic census data.

**Methods:**

Using the Nigerian health clinic census, we evaluate the intercorrelation between the quantity and the quality of health clinics available across the country. We also examine the correlation between the access to health clinics and health behaviors/outcomes for residents by merging the health clinic census data with data from the demographic and health survey (DHS). The health clinic census data makes it possible to capture the overall geographical allocation of health services across the country as well as their comprehensive relationship with health outcomes.

**Results:**

We find a strong positive correlation between the quality of a health clinic and the quantity and quality of neighboring clinics. The high quality clinics are concentrated in areas where the density of clinics is high, and where more of the clinics around them are also of high quality. We also find that an increase in access to health clinics of high quality that are in close proximity is significantly and positively correlated with an improvement in health behaviors as well as health outcomes. Women who are more disadvantaged benefit more from the access to high quality clinics than others.

**Conclusions:**

Health clinics of good quality are unevenly distributed geographically in Nigeria. The quality of health clinics should be of a level that can support the promotion of recommended health behaviors and achieve improved health outcomes throughout the country. Further studies are necessary to evaluate the optimal distribution of clinics of good quality, given that residents in less populated areas gain a higher marginal benefit from improved access to health service, despite the higher costs of supplying the service in those areas.

## Background

The world has observed substantial progress in reducing child mortality in the past decades; child mortality dropped from 93 deaths per 1000 live births in 1990 to 41 in 2016 [1]. Despite this global progress, the burden of deaths is unevenly distributed geographically; one in 36 infants die within the first month in Africa, while the ratio is 1 to 333 in high-income countries. Although the population in Nigeria accounts for only 2.9% of the world population [2], it has a high prevalence of neonatal mortality, which accounts for 9% of the neonatal deaths in the world [1] and 19% of the total maternal deaths in the world [3].

Health service utilization is associated with improved maternal and child health outcomes in Africa [4]. However, the utilization of health services is limited in Nigeria and progress often lags behind other African countries. For example, the percentage of women visiting a clinic for antenatal care is 61% in Nigeria, while the neighboring country Ghana achieves 96% [5, 6], despite the similar level of GDP per capita [7]. The percentage of births assisted by a skilled birth attendant is merely 35.2% in Nigeria, while it is 70.8% in Ghana [8], and the immunization rate for the third dose of the Diphtheria-Tetanus-Pertussis (DTP) vaccine (DTP3) in Nigeria is 33% while Ghana has achieved 99% [9].

Barriers to access to health care in developing countries have been extensively studied. Based on Peters et al. [10] and Ensor and Cooper [11], there are conceptually four dimensions of barriers: geographic accessibility, availability, affordability, and acceptability. Each dimension is found to be important in Nigeria and more broadly in sub-Saharan Africa [12]. Barriers related to geographic accessibility involve problems with access to the service location, such as transportation costs and the lack of means of transport [13–15]. Barriers to availability relate to the lack of a treatment that meets the need of patients, such as drug stockouts, and lack of qualified health workers [16]. Examples of problems with affordability include high service fees and low household incomes, which prevent people from visiting clinics [17, 18], while barriers to acceptability include lack of knowledge and awareness of the importance of health services [19].

We focus on geographic accessibility, one of the four dimensions of barriers to health service utilization. Despite its importance, measuring the effect of accessibility on health service utilization and health outcomes in African countries has been challenging due to the limited availability of data [20–27]. One common approach used to capture the accessibility involves the use of travel time or distance to the clinics typically visited by the respondents, or to the nearest health clinic. However, this approach does not provide insight into the kind of alternative clinics available and reasons why such alternatives are not used. In other words, the lack of complete information on the distribution of health clinics has prevented the comprehensive measurement of the effect of accessibility on health service utilization and health outcomes.

The effect of accessibility on health service utilization can vary greatly based on the quality of the available health facility. The growing literature finds that the quality of the health facility influences health service utilization [28, 29]. However, it has been difficult to obtain information about the quality of health facilities across the country. For example, publicly available health facility data, such as the Service Provision Assessment (SPA), might not contain information about all the health facilities except for some countries, which prevents the accurate measurement of the distance to the health facility in the neighborhood.

Overcoming the above-mentioned measurement challenges using the Nigerian health clinic census, this paper evaluates the interactive effect of access to and the quality of the health facility. As each factor is found to be an important factor in specific settings, it is critical to evaluate its intercorrelation in order to have a comprehensive view on the quality distribution of the health system across the country, as well as estimate its potential impact on the population health. The unique data set of the health clinic census captures the full list of health clinics that exist in Nigeria as of 2013, with geographical information on each clinic. Thus, this data set enables us to construct a comprehensive measurement of the accessibility to and quality of each health facility existent in Nigeria.

We evaluate two research questions. First, we assess the intercorrelation between the quantity and quality of health clinics available within a certain area. We define the quality of a health clinic in terms of the availability of equipment, infrastructure, and services. Then, we evaluate whether health clinics of a certain quality are concentrated within a certain area or spread across a wider region, and if the quality of each health clinic is correlated with the number of health clinics available within its area. This exercise allows us to evaluate the distribution of the available health system. An uneven distribution of high-quality health care, if found to exist, has an important implication on the society. If we find that high-quality health clinics are concentrated in a certain area, this health care disparity can further cause the disparity in health and economic outcomes [30]. Without the health clinic census data, we would not be able to identify if the distribution of health clinics is in fact systematic throughout the country.

Second, we assess the correlation between the access to health clinics and health behaviors and outcomes for residents within catchment areas of health clinics by merging the health clinic census data with the demographic and health survey data (DHS, 2013). Through this exercise, we can test if the access to a high quality facility is positively correlated with improved outcomes. The availability of GPS (Global Positioning System) coordinates for the location of each health clinic, as well as for the cluster where each respondent resides, makes it possible to merge these data sets. Without the census data, it would not be possible to capture the whole picture for the supply side of the health service or the total number of health clinics available within a neighborhood, which is potentially an important determinant of health behaviors and outcomes.

In evaluating the effect of accessibility to health facilities in Nigeria, it is necessary to note the nature of the health care system and the allocation of health facilities in the country. In Nigeria, the health care system is made up of three levels: primary, secondary, and tertiary, depending on the level of services required by patients. The provision of primary health care (PHC) services is the responsibility of the local government, while the secondary healthcare services are under the state government, and the tertiary healthcare services under the federal government [31]. PHC clinics are the first point of contact for patients, who are then referred to the higher-level secondary and tertiary facilities depending on the level of treatment required [32]. The tertiary health care facilities are limited to a maximum number of six per state [33]. The higher-level and private facilities are mostly located in the state capitals or densely populated urban areas [34].

## Methods

### Data

For the analysis, we use two data sets. The first data set is the health facility census data, which captures all the clinics in Nigeria. We use the data publicly available online from the Nigeria MDGs (Millennium Development Goals) Information System (NMIS) health facility database (2014). The data has a complete list of health facilities existing in Nigeria in 2013. The database includes 34,120 clinics, each of which has valid location information. The NMIS health facility database contains information for each clinic relating to location, facilities and services provided, and personnel employed. The location information includes state, local government area (LGA), and GPS coordinates. The information about each clinic’s facilities includes details about whether each clinic has a water supply, toilet, electricity, and a refrigerator or freezer for vaccines. The information on the services provided at each clinic includes antenatal care, family planning, malaria treatment, child delivery service, emergency transport service, skilled birth attendant, C-section service, and measles immunization. The personnel information includes the number of community health extension workers (CHEWs), nurses, and doctors. Out of 34,120 clinics, we use about 74% (25,378 clinics) for the main analysis. We omit the remaining 26% of the clinics due to missing values in one or more of the main variables.

The second data set that we use is collected by the Nigeria Demographic and Health Survey (DHS) conducted in 2013 [35]. The data contains various pieces of information on respondents, including GPS coordinates of the clusters where the respondents reside, health behaviors among the respondents such as antenatal care visits, institutional delivery, postnatal care and vaccination, and health outcomes such as the height and weight of the respondents’ children.

### Measurement of quantity and quality of care

The main explanatory variable is the quantity of health clinics. From the health clinic census, we can observe the entire distribution of health clinics across the country. Using the GPS coordinates of each health clinic, we calculate the distance between a health clinic (let’s call it *A*) and all the other clinics in Nigeria, then calculate the number of health clinics available within a certain distance (we use a radius of 5 km from health clinic *A*). We repeat this exercise for all the health clinics to get the total number of health clinics within a radius of 5 km of every health clinic.

Another main explanatory variable is the quality of health clinics. Evaluation of the quality of care is ongoing. Following extant studies and recommendations [3, 36–40], and in response to the limited information on each health facility available in the census data, we construct a service index based on the service availability and infrastructure to proxy the quality of each clinic. We constructed the index following the broad idea from the WHO SARA Reference Manual [5, 6], by simply combining the components of infrastructure and availability of facilities and services with equal weights assigned to each component [40]. In particular, the quality of a clinic is determined by whether it has the following elements: 1) improved water supply; 2) improved sanitation; 3) electricity; 4) refrigerator/freezer for vaccines; 5) antenatal care service; 6) family planning service; 7) malaria treatment service; 8) emergency transport service; 9) child delivery service; 10) skilled birth attendant; 11) caesarian-section service; and 12) measles vaccination service. As we have 12 components, the service index takes the value of 0 to 12.

This service index, however, does not capture multiple aspects of quality, due to the data limitation. Donabedian [41] described the quality of care using three categories: structure, process, and outcome. Our service index only captures the structure, but not the process and outcome. The index also does not capture the perspectives and perceptions of users and patients, despite their potential importance.

Because the service index focuses exclusively on the structure of the health facility by counting the number of available services, the index is expected to be closely correlated with the level of the health facility. Indeed, we confirm from our data that the service index is positively correlated with the type of facility; hospitals have higher scores on the service index compared to the other types of facilities.

Using this index, we then divide clinics into three categories, low, middle, or high, according to their quality. If the total number of services available is 4 or less, out of 12, the quality of the clinic is categorized as low. If the number is more than 4 but less than 8, it is categorized as medium quality, and if the number is more than 8, it is categorized as a high quality clinic. We further divide the medium quality clinics into high-mid quality (6 < = service index <=8) and low-mid quality (4 < = service index <=5) for some regression to have an even distribution of the clinics in each quality category.

In our analysis, we control for population (2006) and land area in square kilometers for each LGA because we expect the population size and population density of the location to influence the quantity and quality of health clinics.

### Statistical analysis

To evaluate the intercorrelation between the quantity and the quality of health clinics available within a certain area, we employ the simple ordinary least squares (OLS) regression to see the correlation between the number of health clinics available within 5 km of health clinic *i* and the quality of health clinic *i* in the following regression framework:

1 $$ {y}_{ij}=\alpha +{\beta}_1 Num\  Health\ Clinics\ 5{km}_{ij}+{v}_j+{\varepsilon}_{ij} $$

where *y*_*ij*_ is a variable that indicates the quality of health clinic *i* such as the number of services available and number of health professionals (medical doctors, nurses, and community health extension workers) in LGA *j*; *Num Health Clinics* 5*km* indicates the number of health clinics within a 5 km radius of health clinic *i*. As environmental factors such as population density can greatly influence the type of health clinics established in a certain area, we control for LGA fixed effects (v) and cluster standard errors by LGA. LGA fixed effects are important to control for any observed and unobserved factors that can be correlated with the outcomes, such as population density and urban-ness of the area. To examine whether the health clinics of a certain quality are concentrated within a certain area, we add the proportion of health clinics that are of good quality to the regression (1).

We also evaluate the correlation between the quality of a health clinic and the quantity and quality of neighboring health clinics using a different specification:

2 $$ {y}_{ij}=\alpha +\sum \limits_{k\ }{\beta}_{1\_k}{\left( Quality=k\right)}_{ij}+{\beta}_2 Num\  Health\ Clinics\ 5{km}_{ij}+{v}_j+{\varepsilon}_{ij} $$

where *y*_*ij*_ is a variable that indicates the quantity of health clinics within 5 km of the health clinic *i*; (*Quality* = *k*)_*ij*_ is a dummy variable that indicates the quality of health clinic *i* where *k* represents the different levels of quality—high, high-mid, and low-mid. The low quality health clinic is the comparison group in the regression.

Lastly, we evaluate the correlation between the access to health clinics and health behaviors and outcomes for residents within catchment areas of the health clinics. We define the access to health clinics as the number of clinics available within a certain distance from where the respondent resides. We construct this access measurement by merging the health facility census and the DHS, using GPS coordinates of health facilities and respondents in the DHS. Our analysis does not utilize the distance from a respondent’s residency to the nearest health facility because the GPS coordinates for each respondent in the DHS data is perturbed [42]. Rather, we use the number of available clinics in the neighborhood because this method is less sensitive to the measurement bias of the household location in the DHS, similar to the approach adopted in other studies [40]. Then, we estimate the correlation in the following regression framework:

3 $$ {y}_{mn}=\alpha +\sum \limits_{k\ }{\beta}_{1\_k}\%{\left( Quality=k\right)}_{mn}+{\beta}_2 Num\  Health\ Clinics\ 5{km}_{mn}+X{\prime}_{mn}\mu +{v}_n+{\varepsilon}_{ij} $$

where *y*_*mn*_ is a variable that indicates health behaviors and health outcomes, such as antenatal care visits, institutional delivery, postnatal care, vaccination completion, and stunting, for a woman/child *m* in a state *n*; %(*Quality* = *k*)_*mn*_ is a variable that indicates the percentage of health clinics with quality = *k* within 5 km from where the person *m* resides. *Num Health Clinics* 5*km* indicates the number of health clinics within 5 km of a respondent *m*. Other covariates included in X are baseline characteristics and behavior such as wealth index, women’s education level, marital status, height, age, number of household members, number of household members under 5 years old, and location of residence (rural/urban). Similar to the regression framework above, we also control for LGA fixed effects (v) and cluster standard errors by state.

## Results

### Descriptive statistics of health clinics

Table 1 shows the descriptive statistics of health clinics by zone and highlights the difference in quality of health clinics by zone. Nigeria has six zones: three in the South and another three in the North. The North has 3900 to 6200 health clinics in each zone, while the South as 2700 to 3700 clinics in each zone. Once we take the population into account, the number of clinics per 1000 people ranges from 0.25 to 0.47 in the North and 0.25 to 0.30 in the South. A very high proportion of health clinics are public, with 92% in the North and 80% in the South.

**Table 1:** Descriptive Statistics of Health Clinic Census by Zone

Zone	North central	Northe East	North West	South East	South South	South West
	(1)	(2)	(3)	(4)	(5)	(6)
LGA-level characteristics						
Total number of Health Clinics	5837	3904	6279	2916	2705	3737
Number of Clinics in LGA	74.170	55.603	46.702	49.806	41.016	52.686
# Clinics in LGA/Population*1000	0.466	0.331	0.251	0.295	0.245	0.299
# Clinics in LGA/Population density	0.990	0.993	0.339	0.091	0.244	0.180
Population of LGA (2006)	175,398.893	186,431.946	200,425.755	181,054.100	181,071.164	207,906.442
Land area (km2) of LGA	2142.580	2832.435	1366.534	325.050	912.314	612.571
Population density of LGA (millions/km2)	203.560	149.529	564.485	1136.238	422.857	2156.468
Health Clinic Characteristics						
Public	0.874	0.949	0.944	0.777	0.889	0.753
*Equipment*						
Improved water supply	0.389	0.376	0.420	0.489	0.503	0.551
Improved toilet	0.389	0.392	0.429	0.644	0.623	0.698
Vaccine fridge/freezer	0.139	0.159	0.142	0.269	0.202	0.241
Electricity	0.281	0.215	0.224	0.570	0.539	0.625
*Service*						
Antenatal care	0.758	0.551	0.530	0.860	0.850	0.832
Family planning	0.573	0.473	0.446	0.547	0.722	0.627
Malaria treatment	0.742	0.723	0.706	0.832	0.832	0.851
Maternal health delivery service	0.660	0.468	0.338	0.747	0.699	0.712
Emergency	0.213	0.214	0.199	0.269	0.315	0.405
Skilled_birth_attendant	0.261	0.146	0.118	0.545	0.536	0.532
C-section	0.047	0.045	0.042	0.113	0.082	0.108
Measls immunization	0.732	0.751	0.760	0.761	0.789	0.730
Service Index	5.182	4.511	4.352	6.645	6.692	6.911
*Personnel*						
Number of CHEWs (community health extension workers)	2.273	2.213	1.947	2.053	2.239	1.901
Number of nurses	0.329	0.239	0.190	0.950	0.628	0.807
Number of doctors	0.087	0.050	0.059	0.279	0.244	0.383
Number of clinics in 5 km	8.167	4.544	6.936	27.434	10.773	40.213
Proportion of “good” clinics in 5 km	0.237	0.159	0.137	0.397	0.361	0.461

Notes: The sample includes 25,378 health clinics which has GPS soordinates

Comparing the facilities of each health clinic by region, we find that while less than 40% of clinics in the North have improved water supply, 51.8% of clinics in the South have that facility. Only about 40% of the northern clinics have improved toilet facilities, compared to 65.9% of southern clinics. There are refrigerators or freezers for vaccines in less than 15% of clinics in the North, while 23.8% of clinics in the South have this equipment. 24% of clinics in the North have electricity, a contrast to the 58% of southern clinics that have it.

In terms of available services at clinics, about 55% of clinics in the Northeast and Northwest offer antenatal care, while 84.6% of clinics in the South offer the service. Family planning is offered in about 45% of clinics in the Northeast and Northwest, while it is offered in 63% of southern clinics. Maternal delivery service is offered in only 33.8% of clinics in the Northwest but it is offered at 71.9% of clinics in the South. Skilled birth attendants are present only in 17.7% of the northern clinics, while 53.7% of clinics in the South have these attendants. The C-section service is still uncommon across Nigeria; only 4.4% of the northern clinics offer the C-section service, while it is offered in about 10% of clinics in the South. Measles immunization is offered at about 75% of clinics, regardless of location. Overall, out of 12 scores, health clinics in the Northeast and Northwest have the lowest service index at 4.5 or less. Health clinics in the South have scores higher than 6.6 on average.

We also observe differences in the quantity and quality of personnel across regions. Each clinic has an average of 2 CHEWs, regardless of the zone. Clinics in the North have on average 0.25 nurses and 0.07 doctors, while in the South the average number is 0.8 and 0.3 respectively.

The total number of clinics within 5 km of each clinic varies from 4.5 in the Northeast to 40 in the Southwest. The proportion of high quality clinics in the North is only about 18%, while over 40% of such neighboring clinics in the South are considered high quality based on the service index.

### Distribution of health clinics

**Fig. 1:**
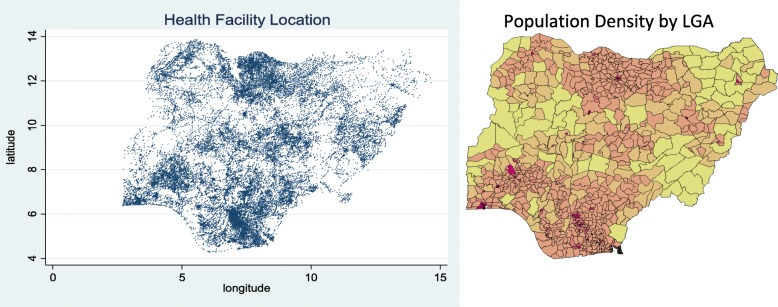
Health Facility Location and Population Density in Nigeria. Source: [Health Facility Location] The author produced the map of health facility location based on health facility census data. [Population Density by LGA] National Population Commission (NPC) [Nigeria] [45]. No permission was required to access and replicate this figure

In this section, we show the geographical distribution and interrelationship of health clinics. Figure 1 shows the distribution of health clinics in Nigeria. We also show the population density by LGA next to the location of a health clinic for comparison. We see a high concentration of both clinics and population in the Southwest—this is where Lagos, the economic center of the country, is. We also see a similar concentration in the northcentral area where Kano, the northern commercial center, is located.

Table 2 (Panel A) shows the correlation between the number of health clinics within 5 km of each clinic and the various indicators of the quality of each clinic. We include the LGA fixed effect to control for any characteristics that are specific to a particular LGA, including population and population density. When the number of clinics within 5 km of a given health clinic increases by 1, the number of services available in that health clinic increases by 0.024. Similarly, the increase in the number of clinics within 5 km increases the number of CHEWs, nurses, and doctors by 0.015, 0.011, and 0.005, respectively.

**Table 2:** Quantity - Quality Relationship of Health Clinics Distribution

	# services available	# CHEWs (Community Health Extension workers)	# nurses	# doctors
*Panel A*	(1)	(2)	(3)	(4)
Number of health clinics within 5 km	0.024*** (0.002)	0.015*** (0.003)	0.011*** (0.002)	0.005*** (0.001)
constant	5.110*** (0.034)	1.883*** (0.037)	0.299*** (0.022)	0.085*** (0.011)
N	25,378	25,378	25,378	25,378
r2	0.014	0.005	0.010	0.007
LGA FE	X	X	X	X
*Panel B*	(1)	(2)	(3)	(4)
Number of health clinics within 5 km	0.016*** (0.002)	0.009*** (0.002)	0.009*** (0.002)	0.004*** (0.001)
Proportion of good quality within 5 km	1.642*** (0.093)	1.180*** (0.099)	0.409*** (0.040)	0.154*** (0.020)
constant	4.786*** (0.033)	1.651*** (0.039)	0.218*** (0.021)	0.055*** (0.010)
N	25,378	25,378	25,378	25,378
r2	0.035	0.015	0.015	0.009
LGA FE	X	X	X	X

Notes: * significant at 10%; ** significant at 5%; *** significant at 1%

Table 2 (Panel B) examines whether, given the number of clinics in a neighborhood, the proportion of high quality clinics in the neighborhood of a health clinic increases the quality of that clinic. We find that if the proportion of high quality clinics is higher around a health clinic, then the number of services available in that health clinic is higher, and the same applies to the number of health personnel. We also examine the correlation by restricting the sample to the same type of clinic, as shown in Table 6, and find a consistent positive correlation, regardless of the type of clinic. Among the same type of clinics, for example PHC clinics, when the number of neighboring clinics increases, the quality of the PHC clinics increases. Similarly, among PHC clinics, when the proportion of neighboring clinics with high quality increases, the quality of PHC clinics increases.

**Fig. 2:**
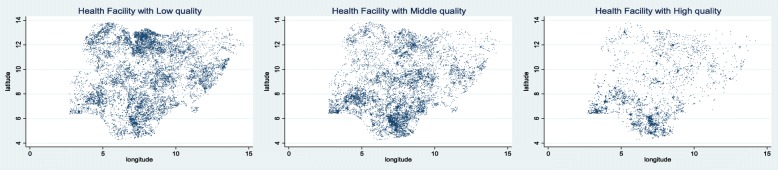
Location of Health Facility by Quality. Source: The author produced the map of health facility location based on health facility census data. Notes: The quality of health facility is determined by the following elements; If the clinic has 1) improved water supply, 2) improved sanitation, 3) electricity , 4) refrigerator/freezer for vaccines, 5) antenatal care service, 6) family planning service, 7) malaria treatment service, 8) emergency transport service, 9) child delivery service, 10) skilled birth attendant, 11) caesarian-section service, 12) measles vaccination service. We divide the health facilities into 3 categories – low, middle, and high quality. If the total number of services available out of 11 service is 4 or less, the clinic has low quality. If it is more than 4 but less than 8, then the clinic has middle quality. If it is more than 8, then the clinic has high quality

Figure 2 depicts the distribution of health clinics in Nigeria according to quality. Low quality clinics are widespread across Nigeria, but we see a high concentration of such health clinics in the northcentral area. High quality clinics, on the other hand, are concentrated in the southern part of the country.

Table 3 shows the correlation between the quality of a health clinic and the quantity and quality of neighboring health clinics. This exercise is similar to what we have shown in Table 2, but we can observe the non-linear relationship between quality and quantity. We show in Table 3 (Column 1) that if a health clinic is of high quality (service index > = 9), the total number of neighboring clinics within 5 km is 5 more than that of a low quality clinic (service index <=3). If a health clinic is high-mid quality (6 < = service index <=8), the total number of neighboring clinics within 5 km is 1.4 more than that of a low quality clinic, while the total number of neighboring clinics within 5 km of a health clinic with low-mid quality (4 < = service index <=5) and with low quality does not change.

**Table 3:** Distribution of Health Clinics

	# health clinics in 5 km	# “good” health clinics in 5 km	Proportion of good quality within 5 km ((2/1))
	(1)	(2)	(3)
(Control: Own Clinic Quality = Low)			
Own Clinic Quality = High	5.029*** (0.499)	3.508*** (0.384)	0.110*** (0.007)
Own Clinic Quality = High-mid	1.362*** (0.221)	0.953*** (0.157)	0.046*** (0.005)
Own Clinic Quality = Low-mid	0.149 (0.177)	0.138 (0.122)	0.019*** (0.004)
Number of health clinics within 5 km			0.004*** (0.001)
constant	13.207*** (0.153)	7.227*** (0.113)	0.164*** (0.008)
N	25,378	25,378	25,378
r2	0.016	0.015	0.086
LGA FE	X	X	X

Notes: The quality of health facility is determined by the following elements; If the clinic has 1) improved water supply, 2) improved sanitation, 3) electricity, 4) refrigerator/freezer for vaccines, 5) antenatal care service, 6) family planning service, 7) malaria treatment service, 8) emergency transport service, 9) child delivery service, 10) skilled birth attendant, 11) caesarian-section service, 12) measles vaccination service. We divide the health facilities into 3 categories – low, middle, and high quality. If the total number of services available out of 11 service is 4 or less, the clinic has low quality. If it is more than 4 but less than 8, then the clinic has middle quality. If it is more than 8, then the clinic has high quality. * significant at 10%; ** significant at 5%; *** significant at 1%

Similarly, if a health clinic is of high quality, then the proportion of health clinics within 5 km that are high quality increases by 11 percentage points compared to the case when a health clinic is of poor quality (Table 3 Column 3). If a health clinic is of high-mid quality, the proportion of neighboring clinics with high quality is 4.6 percentage points more than when a clinic has poor quality. Finally, if a health clinic is of low-mid quality, the proportion of neighboring clinics with high quality is no different from when a clinic is of poor quality.

### Health clinics and health outcomes/behaviors

This section evaluates the correlation between access to a high-quality health clinic and health behaviors/outcomes. Table 4 shows the correlation between the quality of health clinics that are within 5 km of a cluster where mothers and children reside and their health behaviors/outcomes. The main dependent variable is the proportion of health clinics that are considered high quality within 5 km of a cluster. We also evaluate the effect of access to health clinics of different quality: high-middle, low-middle, and low quality.

**Table 4:** Correlation between Quantitiy/Quality of Clinics and Health Outcomes (Individual-level analysis)

	Antenatal care (at least 1)	Antenatal care (at least 4)	Institutional delivery	Postnatal care	Complete Vaccination	HAZ	Stunting
	(1)	(2)	(3)	(4)	(5)	(6)	(7)
% high HF within 5 km	0.243*** (0.023)	0.214*** (0.026)	0.125*** (0.019)	0.054** (0.026)	0.095*** (0.022)	29.258*** (10.451)	−0.046* (0.025)
% high-middle HF within 5 km	0.170*** (0.021)	0.195*** (0.024)	0.052*** (0.018)	0.048** (0.024)	0.047** (0.020)	7.674 (9.612)	−0.004 (0.023)
% low-middle HF within 5 km	0.187*** (0.022)	0.183*** (0.024)	0.038** (0.018)	0.070*** (0.025)	0.014 (0.021)	0.752 (9.988)	0.011 (0.024)
% low HF within 5 km(control)	0.000 (.)	0.000 (.)	0.000 (.)	0.000 (.)	0.000 (.)	0.000 (.)	0.000 (.)
Number of health clinics within 5 km	−0.000 (0.000)	−0.000 (0.000)	0.000 (0.000)	0.001** (0.000)	0.000 (0.000)	0.040 (0.195)	0.000 (0.000)
constant	0.359*** (0.073)	0.090 (0.081)	0.269*** (0.061)	−0.031 (0.083)	−0.059 (0.070)	− 638.953*** (33.522)	1.404*** (0.082)
N	16,988	16,988	25,273	16,928	24,917	22,682	22,681
r2	0.052	0.048	0.060	0.017	0.023	0.017	0.013
LGA FE	X	X	X	X	X	X	X

Notes: Covariates include wealth index, mother’s education in years, marital status, mother’s height, mother’s age, # household members, # household members under 5, rural. The quality of health facility is determined by the following elements; If the clinic has 1) improved water supply, 2) improved sanitation, 3) electricity, 4) refrigerator/freezer for vaccines, 5) antenatal care service, 6) family planning service, 7) malaria treatment service, 8) emergency transport service, 9) child delivery service, 10) skilled birth attendant, 11) caesarian-section service, 12) measles vaccination service. We divide the health facilities into 3 categories – low, middle, and high quality. If the total number of services available out of 11 service is 4 or less, the clinic has low quality. If it is more than 4 but less than 8, then the clinic has middle quality. If it is more than 8, then the clinic has high quality. * significant at 10%; ** significant at 5%; *** significant at 1%

We find that when the proportion of high quality clinics within 5 km increases by 100%, the take-up of at least one antenatal care visit increases by 24.3 percentage points (Table 4 Column 1), and the take-up of at least four antenatal care visits increases by 21.4 percentage points (Column 2). The take-up of institutional delivery, postnatal care, and complete vaccination increases by 12.5, 5.4, and 9.5 percentage points respectively if the percentage of nearby health clinics of high quality increases by 100% (Columns 3 to 5). The access to health clinics of high-middle or of low-middle quality is also positively correlated with health behaviors, but the magnitude of coefficients is smaller and less significant for most of the variables.

We also evaluate the correlation between the access to a health clinic and health outcomes. If the proportion of high quality health clinics within 5 km increases by 100%, the height for age (HAZ) is higher by about 0.29 (Column 6) and the likelihood of a child being stunted is lower by 4.6 percentage points (Column 7). The access to lower quality clinics is no longer significantly correlated with height for age and stunting (Table 4 Column 6).

Other than access to high quality health clinics, other covariates are also correlated with health behaviors/outcomes (Table 7). For example, mothers’ education, marital status, mothers’ health status (height), and the wealth level of the household are positively correlated with better health behaviors and better health outcomes. On the other hand, both the number of children in the household and living in a rural area are negatively correlated with health behaviors. We know that health behavior is influenced both by access to a health facility and the mothers’ socioeconomic status; Table 5 examines the differential effect of access to high quality health clinics based on the mothers’ characteristics, especially education and wealth levels. We observe the differential effect on antenatal care visits. The effect of an increased proportion of high quality clinics in a 5 km radius on antenatal care visits is significantly lower among women with higher education and wealth levels (Table 5 Panel A and B Columns 1 and 2).

**Table 5:** Differential Effect of Quantitiy/Quality of Clinics on Health Outcomes by Mother’s characteristics

	Antenatal care (at least 1)	Antenatal care (at least 4)	Institutional delivery	Postnatal care	Complete Vaccination	HAZ	Stunting
Panel: Education	(1)	(2)	(3)	(4)	(5)	(6)	(7)
% high HF within 5 km (comparison: % low HF within 5 km)	0.284*** (0.025)	0.238*** (0.028)	0.116*** (0.021)	0.044 (0.028)	0.075*** (0.024)	29.507*** (11.234)	−0.050* (0.027)
% high-middle HF within 5 km (comparison: % low HF within 5 km)	0.191*** (0.023)	0.220*** (0.025)	0.049*** (0.019)	0.049* (0.026)	0.045** (0.021)	5.528 (10.119)	−0.001 (0.025)
% low-middle HF within 5 km (comparison: % low HF within 5 km)	0.212*** (0.023)	0.198*** (0.025)	0.046** (0.019)	0.070*** (0.026)	0.011 (0.022)	−6.520 (10.409)	0.017 (0.025)
Education (secondary or higher)	0.221*** (0.025)	0.221*** (0.028)	0.164*** (0.021)	0.085*** (0.028)	0.092*** (0.024)	10.479 (11.120)	−0.050* (0.027)
% high HF within 5 km * Education	−0.171*** (0.032)	− 0.121*** (0.036)	0.029 (0.027)	0.028 (0.037)	0.070** (0.031)	7.253 (14.489)	0.004 (0.035)
% high-middle HF within 5 km * Education	−0.134*** (0.033)	−0.129*** (0.037)	0.012 (0.028)	0.003 (0.037)	0.026 (0.032)	14.774 (14.728)	−0.012 (0.036)
% low-middle HF within 5 km * Education	−0.165*** (0.040)	−0.108** (0.045)	− 0.051 (0.034)	0.003 (0.046)	0.028 (0.039)	44.425** (18.219)	−0.034 (0.044)
constant	0.338*** (0.074)	0.074 (0.081)	0.272*** (0.061)	−0.028 (0.083)	−0.051 (0.070)	− 637.852*** (33.568)	1.404*** (0.082)
N	16,988	16,988	25,273	16,928	24,917	22,682	22,681
r2	0.054	0.049	0.060	0.017	0.023	0.017	0.013
LGA FE	X	X	X	X	X	X	X
Panel B: Wealth	(1)	(2)	(3)	(4)	(5)	(6)	(7)
% high HF within 5 km (comparison: % low HF within 5 km)	0.292*** (0.027)	0.241*** (0.030)	0.101*** (0.023)	0.053* (0.031)	0.063** (0.026)	24.765** (12.365)	−0.064** (0.030)
% high-middle HF within 5 km (comparison: % low HF within 5 km)	0.200*** (0.024)	0.220*** (0.027)	0.050** (0.020)	0.050* (0.027)	0.065*** (0.023)	15.360 (10.909)	−0.020 (0.027)
% low-middle HF within 5 km (comparison: % low HF within 5 km)	0.237*** (0.023)	0.230*** (0.026)	0.074*** (0.019)	0.094*** (0.027)	0.026 (0.022)	0.724 (10.679)	0.012 (0.026)
Wealth = middle, richer, richest	0.233*** (0.021)	0.212*** (0.023)	0.115*** (0.017)	0.091*** (0.024)	0.048** (0.020)	16.566* (9.441)	−0.059** (0.023)
% high HF within 5 km * Wealthy	−0.160*** (0.033)	−0.110*** (0.037)	0.026 (0.028)	−0.016 (0.038)	0.049 (0.032)	4.940 (15.019)	0.047 (0.037)
% high-middle HF within 5 km * Wealthy	−0.123*** (0.031)	−0.100*** (0.034)	− 0.007 (0.026)	−0.017 (0.035)	− 0.039 (0.029)	−17.186 (13.907)	0.048 (0.034)
% low-middle HF within 5 km * Wealthy	−0.223*** (0.035)	−0.206*** (0.038)	− 0.144*** (0.029)	−0.100** (0.039)	− 0.041 (0.033)	1.261 (15.480)	0.005 (0.038)
constant	0.334*** (0.074)	0.069 (0.081)	0.261*** (0.061)	−0.038 (0.083)	− 0.061 (0.070)	− 639.553*** (33.564)	1.408*** (0.082)
N	16,988	16,988	25,273	16,928	24,917	22,682	22,681
r2	0.055	0.050	0.061	0.017	0.024	0.017	0.013
LGA FE	X	X	X	X	X	X	X

Notes: Covariates include wealth index, mother’s education in years, marital status, mother’s height, mother’s age, # household members, # household members under 5, rural. The quality of health facility is determined by the following elements; If the clinic has 1) improved water supply, 2) improved sanitation, 3) electricity, 4) refrigerator/freezer for vaccines, 5) antenatal care service, 6) family planning service, 7) malaria treatment service, 8) emergency transport service, 9) child delivery service, 10) skilled birth attendant, 11) caesarian-section service, 12) measles vaccination service. We divide the health facilities into 3 categories – low, middle, and high quality. If the total number of services available out of 11 service is 4 or less, the clinic has low quality. If it is more than 4 but less than 8, then the clinic has middle quality. If it is more than 8, then the clinic has high quality. * significant at 10%; ** significant at 5%; *** significant at 1%

## Discussion

This paper provides a comprehensive view on the distribution of the health system across the country in terms of the quantity and quality of health facilities available to the population, and its effect on the population health across the country. In particular, this paper evaluates the intercorrelation between the quantity and the quality of health clinics available within a geographical area using health clinic census data in Nigeria. We also evaluate the correlation between the access to health clinics and health behaviors and outcomes for residents within the catchment areas of health clinics.

In general, the North has more health clinics than the South, both in each zone and in the LGAs. It should be noted that the land area of an average LGA is much larger in the North than in the South, while the population of each LGA is similar across Nigeria. As a result, the population density of LGAs in the South is much higher than in the North. Once we take the population into account, the number of clinics per head is similar across Nigeria, although the number is still slightly higher in the North than in the South. Health clinics in the South are, on average, better equipped than those in the North. A similar gap between geographical zones is apparent for services provided at each clinic, but among clinics in the North, the clinics in the Northwest and Northeast provide the fewest services. The number of nurses and doctors, who are more qualified than CHEWs, is significantly higher in the South than in the North. The number of clinics around each clinic shows a large variation by zone. The southern clinics have significantly more clinics within their neighborhoods than the northern clinics. This observation is consistent with the fact that many higher-level clinics and private clinics are concentrated in urban and populated areas [34].

We evaluate the correlation between the quantity of clinics available in a neighborhood and the quality of the clinics. The purpose of the exercise is to observe if there is any relationship between the quantity of health clinics available in an area and the quality of service, based on the service index, offered by each health clinic around the area. Both the quantity and quality of clinics in a certain area can be correlated with the population density in that area. If an area is densely populated, the quantity of clinics in the area is likely to be higher in order to meet the demands of the population. As we define the quality of clinics in terms of service availability, the quality of clinics can also be higher in densely populated areas, similarly to meet high demands. High quality of care is also likely in more densely populated areas because private clinics tend to be located there, and private clinics tend to have better equipment than public ones.

There is a strong positive correlation between the quantity and quality of neighboring health clinics. This result suggests that when there are many clinics available, especially clinics of high quality, the overall quality of clinics in such high-clinic-density areas is high. This positive correlation can be attributed to the high demand for health services due to the large population, leading to the high number of clinics and high quality of clinics. However, this positive correlation between quantity and quality is not only caused by the high concentration of higher-level facilities (such as hospitals) or private clinics in a populated area. Among the same type of clinics, more of them are situated in populated areas and those are more likely to be of high quality.

These results emphasize an uneven concentration of health clinics by quality. People in remote areas, where the population density is low, need to travel a longer distance to reach clinics, which are of lower quality. The implication of the current uneven distribution is that further inequality can be caused in health service utilization and health outcomes between urban and rural residents.

This unequal distribution of health clinics between urban and rural areas in developing countries has been described in the literature [43]. It has been pointed out that a much larger proportion of the rural population is not covered by the health system and has much lower access to health care. Our results contribute to the literature by finding consistent results through a more comprehensive analysis of the distribution of health clinics in Nigeria. To our knowledge, our paper is the first to evaluate the correlation between the quantity and the quality of existent clinics in Nigeria in a comprehensive way [31].

We further evaluate the correlation between the access to clinics of good quality and health behaviors/outcomes. Overall, we find that the access to higher quality clinics is positively correlated with both better health behaviors and improved health outcomes. If the proportion of high quality clinics increases within the neighborhood of a respondent, her health behaviors, such as antenatal care visits, institutional delivery, postnatal care visits, and full vaccination of her children, are on average better than the health behaviors of others who have fewer high quality clinics around. Not only are the health behaviors improved around high quality clinics, but so too are health outcomes, such as height for age and a reduced likelihood of stunting. We also find the differential effect of the access to health clinics of good quality. The access to high quality clinics benefits women with lower education and wealth levels more than women with higher education and wealth levels. In other words, education and wealth can compensate for difficult access to high quality clinics, presumably because these women are empowered to seek better health care and/or can afford transportation costs to high quality clinics that are further away.

Our findings, using comprehensive data and robust methodology, confirm the unequal distribution of health clinics and the negative consequences of the lack of access to health clinics on health outcomes. While the limited access to health care in rural areas is a universal phenomenon due to the costly maintenance of a quality health system in rural areas, which can be equivalent to that in urban areas [44], the quality of health clinics should be kept at a certain level to promote recommended health behaviors and achieve improved health outcomes. Because our results reveal that access to high quality health clinics benefits women who are more disadvantaged than others, the marginal benefit of high quality clinics is higher in a place where many underprivileged women reside. Given that higher marginal benefit of improved access to health services in rural areas, more studies should explore the optimal allocation of resources, namely the quantity and quality of health clinics, in rural areas as compared to that in urban areas.

This study contributes to literature on the impact of access to health clinics in sub-Saharan Africa by using the unique data of the Nigerian health clinic census to evaluate the correlation between the quality and quantity of health clinics as well as the link between the access to health clinics and health behaviors/outcomes. Previous literature linking access to health clinics to health behaviors at the national level has been limited due to limited data availability.

One main limitation is the limited information to capture the multiple aspects of the quality of clinics. This study only captures the service availability, but future studies should explore other dimensions of quality. One other limitation of the study is that the GPS coordinates for each respondent in the DHS data is perturbed. To protect the respondents’ confidentiality, GPS coordinates for each respondent are first aggregated and only the GPS coordinates for the clusters where the respondents reside are shown. Furthermore, the GPS coordinates for each cluster are masked by adding random error. The inaccuracy of GPS coordinates creates an attenuation bias for the estimated coefficient of the quantity and quality of available health clinics on health outcomes/behaviors. Another limitation of the study is the lack of causal interpretation. As the location of the health clinic is likely to be endogenous, this study does not examine the causal relationship between the access to health clinics and health behaviors/outcomes; we only interpret results as the association.

## Conclusions

Using the unique census data on health clinics available in Nigeria, this paper evaluates the intercorrelation between the quantity and quality of health clinics available. We also measure the correlation between the access to health clinics of various qualities and the health behaviors and health outcomes of the population.

This census data on health clinics makes it possible to systematically evaluate the distribution and concentration of health clinics across the country according to their quality, and examine the impact of access to health clinics on health behaviors and outcomes.

We find that the number of other high quality health clinics near any given health clinic is positively correlated with the quality of that health clinic. In other words, if one clinic is found to be of high quality, there will be a high density of high quality clinics in the surrounding area. We also find that if the proportion of high quality clinics near a household increases, then the health behaviors and health outcomes of the household members are both improved.

An important suggestion for future work arises from the study. Currently, high quality health clinics are unevenly distributed geographically. However, the quality of health clinics across the country should promote recommended health behaviors and bring about improved health outcomes. An uneven distribution of clinics can have negative consequences on the population that resides in a location with limited availability of such clinics, especially women who are more disadvantaged. Further studies are necessary to evaluate the optimal allocation of clinics of good quality, given that populations in rural areas gain a higher marginal benefit from improved access to health services, despite the higher costs of supplying the services in such areas.

## Appendix

**Table 6:** Quantity - Quality Relationship of Health Clinics Distribution by Type of Clinic

	# services available	# CHEWs (Community Health Extension workers)	# nurses	# doctors
*Panel A: Sample: Public PHC*	(1)	(2)	(3)	(4)
Number of health clinics within 5 km	0.014*** (0.002)	0.019*** (0.003)	0.002** (0.001)	0.001** (0.000)
Proportion of good quality within 5 km	1.364*** (0.102)	1.404*** (0.129)	0.170*** (0.032)	0.068*** (0.021)
constant	5.470*** (0.031)	1.977*** (0.052)	0.225*** (0.013)	0.055*** (0.007)
N	14,018	14,018	14,018	14,018
r2	0.031	0.027	0.004	0.002
*Panel B: Sample: Public Health Post/Dispensary*	(1)	(2)	(3)	(4)
Number of health clinics within 5 km	0.012*** (0.004)	0.013*** (0.002)	0.003** (0.002)	−0.000 (0.000)
Proportion of good quality within 5 km	0.612*** (0.124)	0.343*** (0.083)	0.020 (0.024)	−0.000 (0.009)
constant	3.246*** (0.035)	1.193*** (0.022)	0.058*** (0.012)	0.024*** (0.004)
N	7638	7638	7638	7638
r2	0.008	0.008	0.002	0.000
*Panel C: Sample: Public Hospital*	(1)	(2)	(3)	(4)
Number of health clinics within 5 km	0.002 (0.020)	0.129*** (0.044)	−0.010 (0.036)	−0.031 (0.027)
Proportion of good quality within 5 km	−0.221 (0.503)	1.511 (0.987)	1.442* (0.840)	0.814** (0.370)
constant	8.032*** (0.276)	0.947 (0.595)	2.219*** (0.484)	1.097*** (0.334)
N	559	559	559	559
r2	0.001	0.073	0.016	0.023
LGA FE	X	X	X	X

Notes: * significant at 10%; ** significant at 5%; *** significant at 1%

**Table 7:** Correlation between Covariates and Health Outcomes (Individual-level analysis)

	Antenatal care (at least 1)	Antenatal care (at least 4)	Institutional delivery	Postnatal care	Complete Vaccination	HAZ	Stunting
	(1)	(2)	(3)	(4)	(5)	(6)	(7)
*Mother’s characteristics*							
Education (secondary or higher)	0.096*** (0.008)	0.121*** (0.008)	0.168*** (0.006)	0.094*** (0.009)	0.127*** (0.007)	25.468*** (3.431)	−0.059*** (0.008)
Married	0.030*** (0.011)	0.049*** (0.012)	0.052*** (0.009)	0.014 (0.012)	0.029*** (0.011)	2.846 (5.050)	−0.008 (0.012)
Mother’s height	0.001*** (0.000)	0.002*** (0.000)	0.000 (0.000)	0.001*** (0.001)	0.002*** (0.000)	3.128*** (0.205)	−0.006*** (0.000)
Age	−0.001* (0.000)	0.001* (0.000)	0.000 (0.000)	0.000 (0.000)	−0.002*** (0.000)	−0.443** (0.200)	0.000 (0.000)
*Household characteristics*							
Wealth index: middle, richer, richest	0.114*** (0.008)	0.113*** (0.009)	0.085*** (0.007)	0.060*** (0.009)	0.036*** (0.008)	12.378*** (3.603)	−0.034*** (0.009)
Number of household members	−0.003*** (0.001)	−0.002* (0.001)	− 0.001 (0.001)	−0.000 (0.001)	− 0.001 (0.001)	−0.603 (0.514)	0.001 (0.001)
Number of household members unde 5 years	− 0.005* (0.003)	−0.008** (0.004)	− 0.015*** (0.003)	−0.006* (0.004)	0.002 (0.003)	1.480 (1.449)	−0.002 (0.004)
rural	−0.106*** (0.013)	−0.108*** (0.015)	− 0.114*** (0.011)	− 0.044*** (0.015)	− 0.047*** (0.013)	−6.105 (5.971)	0.017 (0.015)
Constant	0.359*** (0.073)	0.090 (0.081)	0.269*** (0.061)	−0.031 (0.083)	−0.059 (0.070)	−638.953*** (33.522)	1.404*** (0.082)
N	16,988	16,988	25,273	16,928	24,917	22,682	22,681
r2	0.052	0.048	0.060	0.017	0.023	0.017	0.013
LGA FE	X	X	X	X	X	X	X

Notes: The quality of health facility is determined by the following elements; If the clinic has 1) improved water supply, 2) improved sanitation, 3) electricity, 4) refrigerator/freezer for vaccines, 5) antenatal care service, 6) family planning service, 7) malaria treatment service, 8) emergency transport service, 9) child delivery service, 10) skilled birth attendant, 11) caesarian-section service, 12) measles vaccination service. We divide the health facilities into 3 categories – low, middle, and high quality. If the total number of services available out of 11 service is 4 or less, the clinic has low quality. If it is more than 4 but less than 8, then the clinic has middle quality. If it is more than 8, then the clinic has high quality. * significant at 10%; ** significant at 5%; *** significant at 1%

## Abbreviations

CHEW: Community Health Extension Worker
DHS: Demographic and Health Survey
DTP: Diphtheria-Tetanus-Pertussis
GPS: Global Positioning System
LGA: Local Government Area
MDG: Millennium Development Goal
NMIS: Nigeria MDGs Information System
OLS: Ordinary Least Squares
PHC: Primary Health Care
SPA: Service Provision Assessment
UNICEF: United Nations Children’s Fund
WHO: World Health Organization
